# High flow nasal cannula versus noninvasive ventilation in the treatment of acute hypercapnic respiratory failure: A systematic review and meta‐analysis

**DOI:** 10.1111/crj.13695

**Published:** 2023-09-12

**Authors:** Aisling C. Fahey, Martina O'Connell, Nicola Cornally, Mohamad M. Saab

**Affiliations:** ^1^ Catherine McAuley School of Nursing and Midwifery University College Cork Cork Ireland; ^2^ Kerry Mental Health Services, Cork/Kerry Community Healthcare Health Service Executive Tralee Co. Kerry Ireland; ^3^ Health Service Executive National eRostering Project, Community Healthcare Operations Improvement and Change St. Loman's Hospital Lucan Co. Dublin Ireland

**Keywords:** acidosis, blood gas analysis, hypercapnia, meta‐analysis, noninvasive ventilation, oxygen saturation, respiratory, respiratory insufficiency

## Abstract

Chronic obstructive pulmonary disease can lead to acute hypercapnic respiratory failure (AHRF), often treated using noninvasive ventilation (NIV). Emerging research suggests the potential utility of high flow nasal cannula (HFNC) for AHRF. This systematic review and meta‐analysis aimed to determine the effect of HFNC versus NIV on AHRF management. A search of electronic databases (CINAHL, MEDLINE, and Academic Search Complete), web sources, and trial registries was last conducted on 9 February 2023. Quality and risk of bias assessments were conducted. Meta‐analyses were used to synthesise data. Seven randomised controlled trials were included. No statistically significant differences between HFNC and NIV were found within the following outcomes of interest: (i) correction of pCO2: standardised mean difference (SMD) = −0.16, 95% confidence interval (CI) (−0.34 to 0.02), *p* = 0.08; (ii) correction of pH: SMD = −0.05, 95% CI (−0.25 to 0.14), *p* = 0.59; (iii) correction of pO2: SMD = −0.15, 95% CI (−0.40 to 0.09), *p* = 0.22; (iv) intubation rates: risk ratio (RR) = 0.87, 95% CI (0.41 to 1.82), *p* = 0.71; (v) mortality rates: RR = 0.85, 95% CI (0.47 to 1.56), *p* = 0.61; and (vi) treatment switch: RR = 1.30, 95% CI (0.43 to 3.94), *p* = 0.64. More controlled trials with large sample sizes are required to investigate the management of AHRF of various aetiologies. HFNC may be used as a final exhaustive measure for COPD‐related AHRF where NIV is not tolerated, and when it is not clinically indicated to extend to endotracheal intubation.

## Graphical abstract

We conducted a systematic review and meta‐analysis to determine if high‐flow nasal cannula can be utilised when managing patients with acute hypercapnic respiratory failure, as opposed to noninvasive ventilation which is conventionally used in this regard. We found no statistically significant differences between the two modalities in the correction of arterial blood gases, intubation rates, mortality rates, and treatment switch.

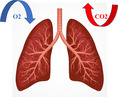

## 1 INTRODUCTION

Acute hypercapnic respiratory failure (AHRF) is a pulmonary condition characterised by insufficient alveolar ventilation leading to hypercapnia (pCO2 > 45 mmHg), hypoxia (pO2 < 60 mmHg) and often, acidosis (pH < 7.35).^1^, ^2^, ^3^ AHRF occurs in approximately 20% of acute exacerbations of chronic obstructive pulmonary disease (COPD) and is associated with increased mortality rates, dependent on the degree of concurrent respiratory acidosis.^4^

Currently, noninvasive ventilation (NIV) is the main treatment modality for AHRF, particularly when complicated by respiratory acidosis.^3^, ^5^ NIV use has been associated with decreased length of hospital stay, improved mortality rates, and decreased requirement for invasive ventilation via endotracheal intubation and is often used as the ceiling of care for individuals where escalation plan does not include invasive mechanical ventilation.^4^, ^6^

NIV is not without drawbacks. NIV failure has been defined as the need for endotracheal intubation due to lack of improvement in arterial blood gas (ABG) values or clinical parameters, clinical deterioration with subsequent endotracheal intubation during period of hospitalisation, or death.^7^, ^8^ NIV intolerance is a common reason for NIV failure in patients with acute and chronic respiratory failure,^9^, ^10^ with issues such as mask‐related claustrophobia, gastric inflation, facial skin breakdown, air leaks, agitation, and patient‐ventilator asynchrony being cited as some of the most frequent causes of interface intolerance which can cause disruptions to therapy.^3^, ^10^, ^11^ Frat et al.^12^ noted the immediate return of hypoxia and work of breathing caused by interruptions to NIV therapy in patients with hypoxemic respiratory failure and suggested that this risk may be mitigated by the use of high flow nasal cannula (HFNC) to facilitate break periods from NIV.

The advantages of HFNC include comfortable nasal interface, increased humidity (which aids mucociliary clearance and reduces nasal dryness), anatomical dead space washout of waste gases including carbon dioxide, decreased respiratory rate, positive end‐expiratory pressure (PEEP) effect (due to high flow rates), and increased tidal volume.^13^, ^14^ HFNC has become a favourable choice in the contemporary management of hypoxemic respiratory failure in adults due to its oxygen delivery capabilities and comfortable interface.^12^, ^15^

Emerging data suggest that the functional benefits of HFNC such as anatomical dead‐space washout, “PEEP‐effect,” enhanced secretion management and comfortable interface may also be advantageous in the management of AHRF.^16^, ^17^, ^18^, ^19^ The aim of this systematic review and meta‐analysis was to determine the effect of HFNC compared to NIV on the management of AHRF. The following primary and secondary objectives were explored, in line with the concept of treatment failure:

Primary objective:

- The effect of HFNC compared to NIV on correcting ABG abnormalities (pCO2, pH, pO2) in individuals with AHRF.

Secondary objectives:

- The effect of HFNC compared to NIV on intubation rates among individuals with AHRF.
- The effect of HFNC compared to NIV on mortality rates among individuals with AHRF.
- The risk of needing to switch to the opposite arm of treatment (e.g., HFNC to NIV and vice versa) among individuals with AHRF.

## 2 METHODS

This systematic review and meta‐analysis was guided by the Cochrane Handbook for Systematic Reviews of Interventions^20^ and reported using the Preferred Reporting Items for Systematic Reviews and Meta‐Analyses (PRISMA) checklist.^21^

### 2.1 Eligibility criteria

Using the population, intervention, comparison, outcome(s) (PICO) Framework,^22^ the review inclusion criteria were as follows: population: adults (≥18 years of age) with AHRF regardless of the underlying diagnosis; intervention: HFNC; comparison: NIV; primary outcome: abnormalities in arterial pCO2, pH, and pO2; and secondary outcomes: intubation rates, mortality rates, and the risk of needing to switch to the opposite arm of treatment (i.e., HFNC to NIV and vice versa). Only randomised controlled trials (RCTs) were included. Studies with participants who have chronic or stable (i.e., non‐acute) hypercapnic respiratory failure were excluded (Table S1).

### 2.2 Search strategy

Subject headings were identified and a single search strategy was devised using three key concepts namely: AHRF, HFNC, and NIV (Table S1). The search was conducted in CINAHL, MEDLINE, Academic Search Complete, Google Scholar, ClinicalTrials.gov, EU Clinical Trials Register, WHO International Clinical Trials Registry Platform, and The Cochrane Central Register of Controlled Trials. Citations from relevant systematic reviews and meta‐analyses were scrutinised. The search was conducted within title or abstract fields. Records were searched from inception to 9 February 2023.

### 2.3 Study selection and extraction

Records were imported into Zotero citation^23^ management software and then exported to Covidence,^24^ an online programme for systematic review record screening. Duplicates were deleted automatically. Two reviewers independently screened the titles and abstracts of all records. The full text of potentially relevant studies was then reviewed by both reviewers, and conflicts were resolved following discussion.

Data from the included studies were extracted into a table adapted from Fineout‐Overholt et al.^25^ under the following headings: author(s); year; country; aims and objectives; design/methods; sample/setting; data collection tools; interventions; outcome; and limitations (Table S2). Data were extracted by the first reviewer and a sample of extracted studies was shared with a second reviewer for cross‐checking.

### 2.4 Data synthesis

The Cochrane Handbook guidance on undertaking meta‐analyses^26^ and RevMan software version 5.4^27^ were used to perform meta‐analyses. Meta‐analyses were conducted according to the systematic review outcomes. Study effect sizes were pooled as risk ratios (RR) for dichotomous outcomes with a confidence interval (CI) of 95%, and the Mantel–Haenszel method for statistical analysis was used as it is preferred in instances where data are sparse,^26^ which was the case in the present review due to the small sample sizes in the included studies.

Continuous outcome data were summarised using means and standard deviations (SD). Wan et al.'s^28^ formula was used to estimate the mean and SD in studies where the median and interquartile range (IQR) were reported. Inverse‐variance weighting was used for statistical analysis and effect sizes were presented as standardised mean difference (SMD) with 95% CI. SMD was used to account for the variability of data‐reporting formats (i.e., mean and SD, and median and IQR).

Heterogeneity was presented using *p*‐value and *I* ^2^. A *p* value of 0.10 was considered statistically significant when dealing with heterogeneity in the meta‐analyses, in keeping with Cochrane guidance for studies with small sample sizes.^26^ Interpretation of statistical significance within the included studies remained at *p* = 0.05, as per normal convention.^26^ A random effects model was used to incorporate statistically significant heterogeneity into the meta‐analysis.

### 2.5 Quality and risk of bias assessments

The methodological quality of the included studies was appraised using the Joanna Briggs Institute critical appraisal tool for RCTs.^29^ The Cochrane RoB 2 tool^30^ was used to assess the risk of bias in RCTs. Traffic‐light plots were generated using the Robvis web‐application.^31^ Quality and risk of bias assessments were conducted by the first reviewer and were cross‐checked by a second reviewer.

## 3 RESULTS

### 3.1 Study selection

**FIGURE 1:**
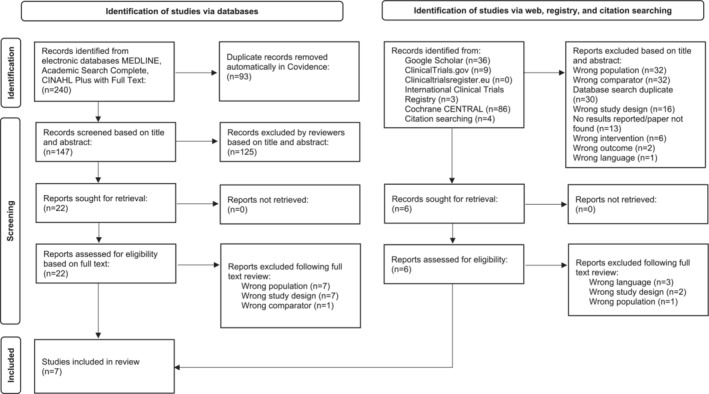
Study identification, screening, and selection process.

A total of 240 records were identified from the databases. Following deletion of duplicates, the titles and abstracts of 147 records were screened. Of those, 22 were sought for full text screening and seven were included in this review. Web, registry, and citation searching yielded 138 records. A total of 132 records were eliminated after titles and abstract screening. Six records were sought for retrieval and were subsequently excluded. Therefore, a total of seven studies were included in the present review (Figure 1).

### 3.2 Study characteristics

Of the seven included studies, the majority were conducted in Asia (China [*n* = 3] and Iran [*n* = 1]). Sample size ranged from 30^32^ to 168 participants.^33^ The duration of the intervention (i.e., HFNC) was inconsistent across the included studies. For example, Jing et al.^34^ used HFNC or NIV continuously for an 8‐h period without interruption, whereas in other studies, patients also received the intervention intermittently. For instance, Rezaei et al.'s^32^ participants received both HFNC and NIV for 30 min, followed by a 1‐h washout period, before switching to the other intervention (i.e., HFNC to NIV or vice versa).

As per the predetermined inclusion criteria, this review intended to study the effect of HFNC on individuals with AHRF regardless of the underlying diagnosis. The included RCTs predominantly focused on patients with COPD, with the exception of one study,^35^ which also included individuals with AHRF secondary to congestive heart failure. All seven studies reported on ABGs, and the majority (*n* = 4) collected data pertaining to physiological parameters such as SpO2, respiratory rate, and heart rate (Table 1).

**TABLE 1:** Study characteristics (*n* = 7).

Country	China (*n* = 3) United States (*n* = 1) Greece (*n* = 1) Iran (*n* = 1) Italy (*n* = 1)
Study setting^a^	Emergency department (*n* = 4) Intensive care unit (*n* = 4) Respiratory ward/unit/centre (*n* = 3)
Sample size (min–max)	30–168
Background diagnoses^b^	Chronic obstructive pulmonary disease (*n* = 7) Concurrent acidosis (*n* = 4) Cardiogenic pulmonary oedema/congestive heart failure (*n* = 1) Post‐extubation (*n* = 2)
Data collected	Physiological parameters and arterial blood gas values (*n* = 4) Physiological parameters, arterial blood gas values, and work of breathing score (*n* = 2) Arterial blood gas values, comfort, and satisfaction levels (*n* = 1)
Follow‐up times (min–max)	150 min to 5 days

^a^ Three studies took place across more than one setting.

^b^ Three studies evaluated patients with a variety of diagnoses.

### 3.3 Quality and risk of bias assessments

Outcome assessors were blind to treatment assignment in three RCTs.^32^, ^36^, ^37^ This was unclear in the remaining four RCTs.^33^, ^34^, ^35^, ^38^ Blinding of participants and those delivering treatment was not possible as these treatment techniques are widely used and easily recognisable in clinical practice; therefore, these criteria were rated as not applicable. Outcome measures and statistical analyses were each consistently reliable and appropriate in all seven RCTs (Table S3).

**FIGURE 2:**
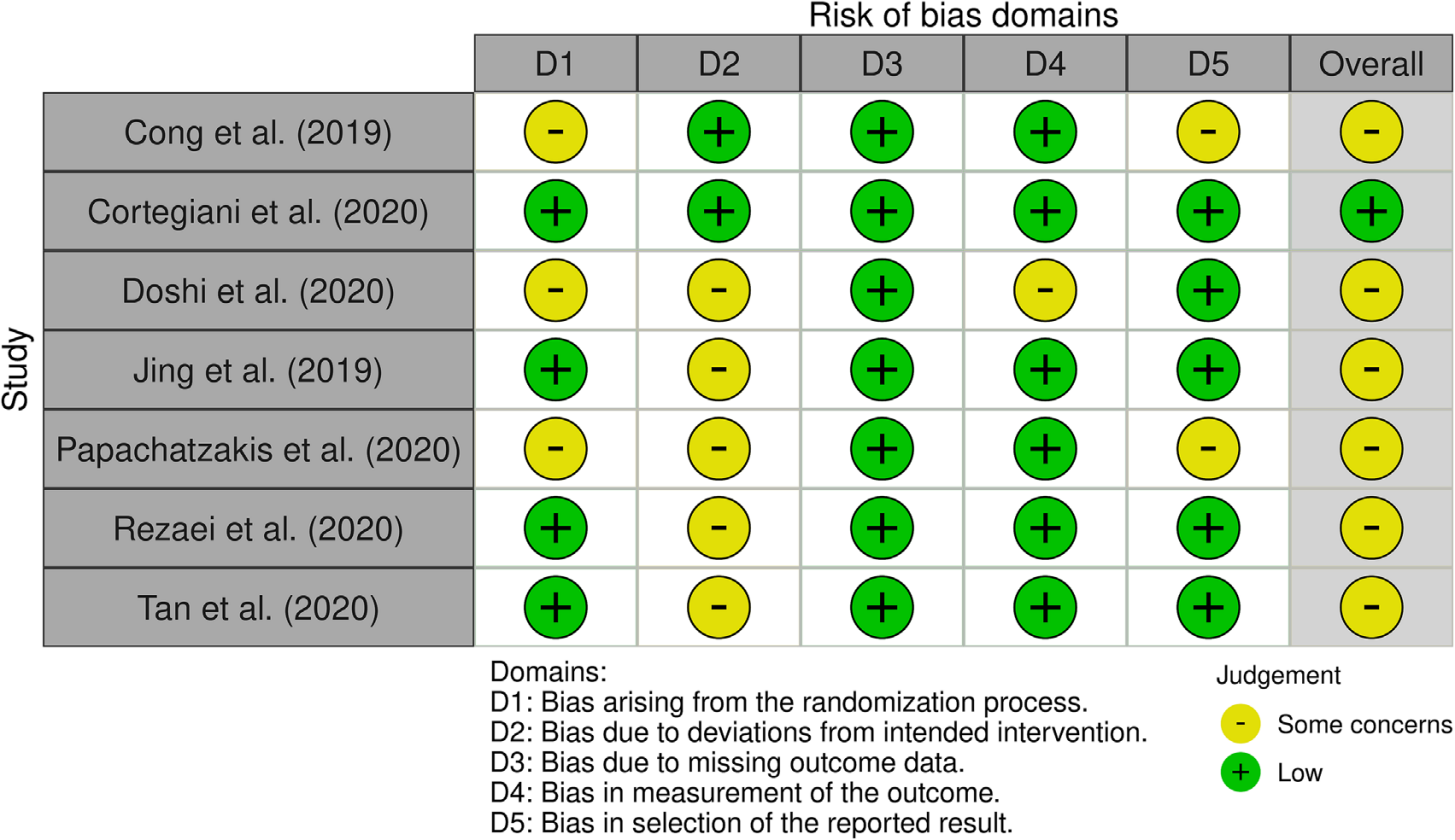
Risk of bias assessment of randomised and nonrandomised controlled trials using the RoB 2 tool and ROBINS‐I tool, respectively.

With the exception of Cortegiani et al.,^36^ which was graded as low risk of bias, RCTs (*n* = 6) were graded as “some concerns.” This predominantly related to participant and caregiver blinding, as devices used for intervention and comparison in the included studies are likely to be identifiable to caregivers and study participants (Figure 2).

### 3.4 Synthesis of results

Statistically significant findings are summarised in Table 2. Findings from individual studies are presented in Table S4. In RCTs, where the per‐protocol analysis and intention‐to‐treat analysis were available, the per‐protocol data were included in the meta‐analysis, as the primary function was to demonstrate the effect of adhering to the intervention (i.e., HFNC vs. NIV in the management of AHRF). The timepoints at which outcomes were measured throughout the included studies varied; therefore, the final measurement provided for each outcome was used for meta‐analysis.

**TABLE 2:** Visual representation of key outcomes.

Author(s) and country		Outcomes				
	pH	pCO2	pO2	Intubation	Mortality	Treatment switch
Cong et al.^33^	X	X	X	NR	NR	NR
Cortegiani et al.^36^	NR	X	NR	X	X	✓†
Doshi et al.^38^	X	✓	X	X	NR	X
Jing et al.^34^	✓	X	NR	X	X	NR
Papachatzakis et al.^35^	X	X	X	NR	X	X
Rezaei et al.^32^	X	X	NR	NR	NR	NR
Tan et al.^37^	X	✓‡	NR	X	X	X

*Note*: ✓ = statistically significant difference in effect between interventions. X = no statistically significant difference in effect between interventions. NR = not reported.

^†^ Statistically significant switch rate in both intervention groups.

^‡^ Statistically significant result for effect of HFNC but not between interventions.

#### 3.4.1 Primary outcome: arterial blood gas values

- pCO2

All studies (*n* = 7) reported changes to pCO2 measurements. The timepoints at which results were reported varied widely from 30 min,^32^, ^38^ to 5 days.^33^ Therefore, meta‐analysis was conducted for data reported at the final point of each study, and overall effect was the measure of interest. The random effects model was used to account for differences in effect.

The formats used to report data varied; five studies reported the mean and SD^32^, ^33^, ^34^, ^35^, ^36^; and two studies reported the median and IQR.^37^, ^38^ In order to construct a meta‐analysis with as much data as possible, mean and SD values were estimated from median and IQR using the formula devised by Wan et al.,^28^ and SMD was used when constructing forest plots to account for differences among original reported values. A forest plot was constructed using only data originally reported in mean and SD to test sensitivity (Figure S1).

**FIGURE 3:**
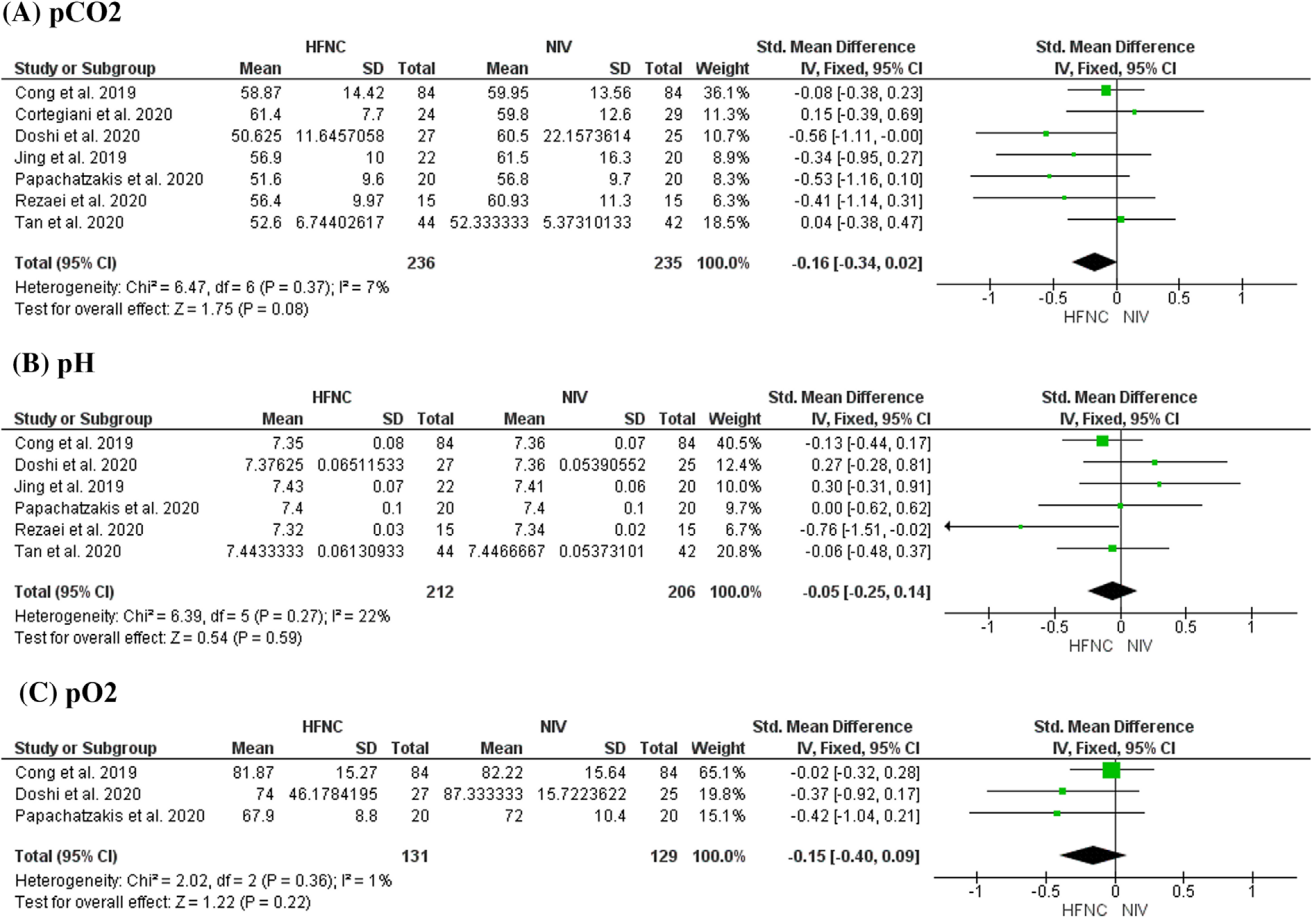
Forest plots for continuous outcomes.

Seven RCTs reported on this outcome and were analysed.^32^, ^33^, ^34^, ^35^, ^36^, ^37^, ^38^ The results were not statistically significant (SMD = −0.16, 95% CI [−0.34 to 0.02], *Z* = 1.75, *p* = 0.08) (Figure 3).

Heterogeneity was present, although not statistically significant (*I* ^2^ = 7%, *p* = 0.37). pCO2 levels were recorded at 24 h in three studies. These data were formed into a forest plot and compared to the original plot depicting final points of each study to test sensitivity (Figure S1).

ii pH

Six studies reported pH values at post‐test. Values recorded at the final timepoint of each study were analysed. The overall effect between the two treatment methods was similar (SMD = −0.05, 95% CI [−0.25 to 0.14], *Z* = 0.54, *p* = 0.59), and not statistically significant. Heterogeneity was present (*I* ^2^ = 2%, *p* = 0.27), although not statistically significant. No further exploration into diversity was conducted and a fixed effect model was used.

iii pO2

Three studies reported pO2 values measured at post‐test. The difference in effect between HFNC and NIV was not statistically significant (SMD = −0.15, 95% CI [−0.40 to 0.09], *Z* = 1.22, *p* = 0.22). Heterogeneity between studies was low (*I* ^2^ = 1%, *p* = 0.36), therefore a fixed effects model was used.

#### 3.4.2 Secondary outcome: intubation rate

**FIGURE 4:**
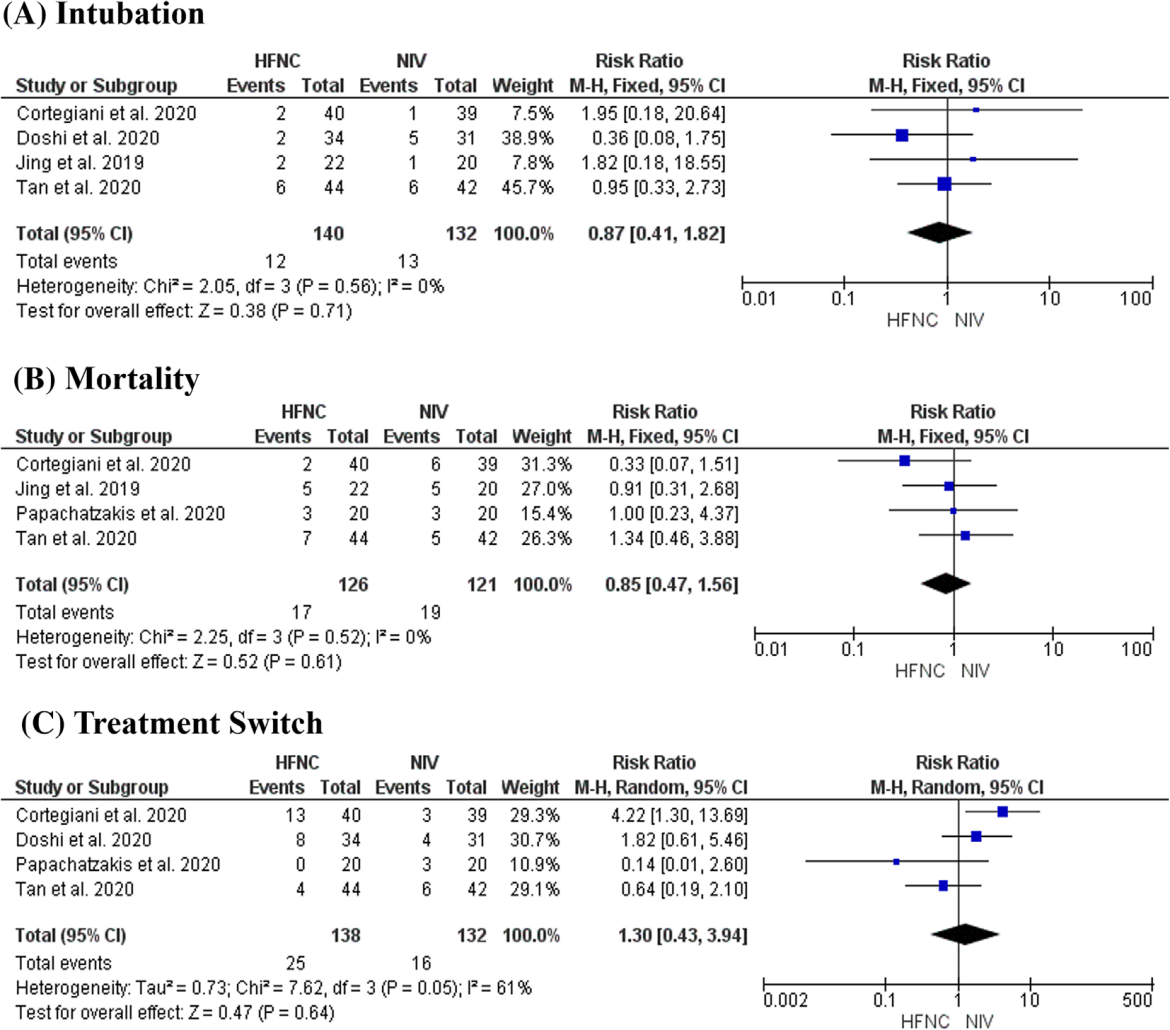
Forest plots for dichotomous outcomes.

Four studies compared the effects of HFNC and NIV on rate of intubation. There was no heterogeneity between the studies (*I* ^2^ = 0%, *p* = 0.56). A fixed effects model was used. The effect was not statistically significant (RR = 0.87, CI 95% [0.41 to 1.82], *Z* = 0.38, *p* = 0.71) (Figure 4).

#### 3.4.3 Secondary outcome: mortality rate

Four studies reported rate of mortality. The time of reporting ranged from recorded in‐hospital mortality,^36^ 28‐day mortality,^34^, ^37^ to 30‐day mortality.^35^ There was no heterogeneity between the studies (*I* ^2^ = 0%, *p* = 0.52). A fixed effects model was used. Overall effect measured between HFNC and NIV was not statistically significant (RR = 0.85, 95% CI [0.47 to 1.56], *Z* = 0.52, *p* = 0.61) (Figure 4).

#### 3.4.4 Secondary outcome: treatment switch

Four studies reported on the incidence of patients switching to the opposite intervention. The degree of heterogeneity (*I* ^2^ = 61%) was moderate to substantial.^20^ This was statistically significant (*p* = 0.05); therefore, a random effects model was used. The overall effect favoured NIV, suggesting a lower risk of switching from NIV to HFNC (RR = 1.30, CI 95% [0.43 to 3.94], *Z* = 0.47), and therefore a higher risk of being switched from HFNC to NIV, although this effect was not statistically significant (*p* = 0.64) (Figure 4).

## 4 DISCUSSION

Findings of the meta‐analysis favoured HFNC for correction of abnormalities in pCO2, pH, and pO2, as well as mortality rates and risk of intubation, although none of these findings were of statistical significance. Risk of switching to the opposite intervention was found to be higher among the HFNC group, although again, this finding was not statistically significant, and given the degree of heterogeneity (*I* ^2^ = 61%; *p* = 0.05), should be approached with caution. A similar review conducted by Ovtcharenko et al.^39^ investigated the potential use of HFNC versus NIV in the management of AHRF by evaluating its impact on mortality, endotracheal intubation, hospital and intensive care unit length of stay, comfort, dyspnoea, respiratory rate, pO2, and pCO2 within eight RCTs and concluded that the current body of evidence is limited in determining whether HFNC is superior, inferior, or equivalent to NIV in the management of AHRF. While the present review does also cite several of the same trials, it does also assess other outcomes such as changes in arterial pH and pO2, as well as risk of switching from HFNC to NIV, and vice versa.

Within the present review, none of the outcomes of interest had overall effect sizes of statistical significance. All outcomes were subject to meta‐analysis. Observing the effect sizes within each meta‐analysis, it appears that for ABGs (i.e., pH,^32^, ^33^, ^34^, ^35^, ^37^, ^38^ pCO2,^32^, ^33^, ^34^, ^35^, ^36^, ^37^, ^38^ and pO2^33^, ^35^, ^38^), the literature favours HFNC; however, this finding is not of statistical significance. Findings pertaining to the secondary outcomes of intubation^34^, ^36^, ^37^, ^38^ and mortality^34^, ^35^, ^36^, ^37^ also favour HFNC, while treatment switch^35^, ^36^, ^37^, ^38^ rates favoured NIV in meta‐analysis. However, as with the findings of meta‐analysis of continuous outcomes; results were also not of statistical significance. Four RCTs tested the alternative hypothesis that HFNC was noninferior to NIV in terms of their own predetermined definitions of clinical efficacy.^32^, ^34^, ^36^, ^37^ Doshi et al.^38^ conducted a subgroup analysis of their noninferiority trial testing HFNC (intervention group) versus NIV (control group) in undifferentiated respiratory failure, so despite the favourable outcomes of the intervention group in this study, the methods used cannot support any strong recommendations for the use of HFNC in AHRF. The remaining studies were less explicit in their hypotheses, although, final recommendations/conclusions can be seen in Table S5. The consensus is that HFNC has similar clinical efficacy and is a potential alternative to NIV within the sample of interest, particularly if NIV is poorly tolerated.

Moretti et al.^7^ refer to “treatment failure” when discussing NIV as the need for intubation due to insufficient improvement in arterial blood gas measurements in the initial hours, clinical deterioration and subsequent intubation during hospital admission, and patient mortality. To cover the spectrum of this definition, this systematic review and meta‐analysis used ABG measurements, intubation rates and mortality rates of the control group (NIV) and the intervention group (HFNC) as the outcomes of interest.

Initially, “treatment switch” was selected as an outcome to indicate the rate of intolerance of either HFNC or NIV. However, the term “treatment switch” was not exclusively used to describe intolerance. For instance, in one study, treatment switch was due to patient discomfort/intolerance,^35^ whereas in all other studies reporting treatment switch (*n* = 3), the reason was a combination of intolerance of the treatment and worsening patient condition/lack of improvement.^36^, ^37^, ^38^ Doshi et al.^38^ noted that the differences in rates of treatment switch between the intervention and control groups may suggest that clinicians may simply be more familiar with NIV. A potential explanation for the heterogeneity in the meta‐analysis of this outcome could be that clinicians switched treatments more readily from HFNC to NIV because they were more confident in the effects of NIV. In this case, the lack of clinician blinding may have introduced bias that influenced study outcomes. Caregiver blinding is unlikely to be possible in future similar studies, meaning there is always potential for performance bias. This could be mediated (although not remedied) by data collector and outcome assessor blinding and consensus with a second, blinded clinician before altering treatment interventions.^40^, ^41^ Despite the frequent pooling of treatment switch secondary to intolerance and clinical deterioration, some studies^35^, ^37^ reported statistically significant differences in the rates of patient tolerance of each interface, with results favouring HFNC. Millar et al.^42^ report a case study in which a patient with hypercapnia and concomitant acidosis who refused to be treated with NIV was administered HFNC, resulting in resolution of acidosis and acceptable improvement in hypercapnia in the context of her underlying COPD. Despite the heterogeneity within the outcome regarding treatment switch, the available evidence favours HFNC in terms of patient tolerance.

Hypercapnia secondary to COPD is well represented within the included literature, featuring in all (*n* = 7) of the included studies. Faqihi et al.^43^ note the scarcity of literature regarding NIV intervention in AHRF outside of COPD and cardiogenic pulmonary oedema, particularly in relation to common conditions such as obesity hypoventilation syndrome. Golmohamad et al.^44^ also make recommendations against the use of HFNC in the management of patients with obesity hypoventilation syndrome/sleep disordered breathing, owing to the lack of research in the area and the incidence of treatment failure in this patient subgroup within their observational study. Considering the various aetiologies of AHRF that often require NIV,^3^ it would be of value to see HFNC used in the context of hypercapnia associated with conditions beyond COPD.

Similarly, systematic reviews have been conducted in relation to the management of hypercapnia in acute exacerbations of COPD using HFNC,^14^, ^45^, ^46^, ^47^ although none examined the effects of this intervention on patients with other background diagnoses. There were also some other differences; not all of these studies exclusively used NIV as the comparator,^14^, ^47^ and Huang et al.^45^ analysed the effect of HFNC and conventional oxygen therapy in comparison to NIV. There is some agreement within the findings of the present review compared with these studies. For example, similar to this review, Yang et al.^47^ found no increased risk of intubation and mortality associated with HFNC in comparison to conventional oxygen therapy. Although this was also not a statistically significant finding, this may be noteworthy. Xu et al.^46^ concluded that HFNC is more advantageous than NIV in the correction of ABG abnormalities and the reduction in the risk of mortality. The present review also observed a similar effect of HFNC on risk of mortality and trends in the correction of blood gas abnormalities.

This meta‐analysis suggests that HFNC may be noninferior to NIV in improving pH, pCO2, pO2, and in terms of risk of mortality and requiring intubation. Mittal et al.^48^ presented a case in which a patient presenting with exacerbation of COPD with hypercapnia and acidosis was treated with HFNC due to intolerance of NIV and lack of consent to intubate. In this case, acute hypercapnia and acidosis were successfully resolved by HFNC. The degree of acidosis would be classified as extreme as per Davidson et al.,^3^ as the patient presented with an arterial pH of 7.23 on room air. HFNC increased arterial pH to 7.33 within 12 h, with equally impressive impact on other abnormal ABG parameters (pO2 and pCO2). Vogelsinger et al.^49^ also observed significant improvement in ABG values in patients with COPD who were treated with HFNC. The literature does call for further research regarding timing of the start, end, and monitoring of therapy.

The timepoints at which outcomes were measured and reported varied widely across included studies. According to Ozyilmaz et al.,^50^ 68% of NIV failures occur within the early stages of treatment (hours 1–48), characterised by inability to correct ABGs and respiratory rate as well as no improvement in subjective reporting of dyspnoea and discomfort,^7^, ^51^ with failures occurring past 48 h being classified as late treatment failure. Similar reviews do not report timepoints at which data were reported which may affect the integrity of conclusions drawn from the data if the timepoints of measurement are vastly different.^14^, ^45^, ^46^ Similar to Yang et al.,^47^ in the present review where multiple timepoints were recorded, the latest recording was used. However, considering the likely clinical significance of the difference in ABG values after 150 min versus 5 days, a separate forest plot to test sensitivity was constructed depicting only pCO2 measurements taken at 24 h (the most commonly occurring timepoint with sufficient data for meta‐analysis) (Figure S1).

One of the included studies was a subgroup analysis of a larger study exploring the impact of HFNC on acute respiratory failure arising in the context of various aetiologies.^38^ The sample sizes of this subgroup is therefore smaller and not selected with the intention of investigating the specific impact of HFNC in AHRF, which may limit generalisability of results with regard to population of interest for this review.

## 5 LIMITATIONS

Some limitations within the included studies are worthy of note. There were some issues with methodological quality and small sample sizes. This may reduce the generalisability of results. The lack of blinding of care providers in the RCTs raises concerns for performance bias. Furthermore, the use of adjunctive therapies such as bronchodilators, corticosteroids, antibiotics, mucolytics, theophylline, or chest physiotherapy were not consistently discussed in the included studies and may have confounded results. The duration of the intervention was inconsistent across all studies. This may challenge the internal validity of results.

The formats of reported statistics were not homogenous enough to be eligible for plotting in meta‐analysis. Two studies reported the median and IQR.^37^, ^38^ Mean and SD were estimated using the formula devised by Wan et al.^28^ A drawback to this practice is that the reporting of IQRs usually indicates the presence of skewed distribution; therefore, these estimates should be approached with caution.^20^

The reviewed literature focused predominantly on COPD as the underlying diagnosis, yet there are various diagnoses associated with AHRF. This highlights the need for future RCTs focused on diagnoses other than COPD. Several RCTs in this area are currently underway. Of particular interest, there is a registered RCT (study completion date: 01 December 2022; not yet published) comparing HFNC and NIV in AHRF secondary to acute cardiogenic pulmonary oedema^52^ and another published RCT protocol, which has potential for a large sample (not fixed; however capped at 2000 participants) examining the impact of HFNC compared to NIV in the management of acute respiratory failure, with a subgroup analysis of individuals with AHRF.^53^

## 6 CONCLUSION

In the context of underlying COPD, HFNC may be as effective as NIV in the management of AHRF, particularly in improving arterial pH, pCO2, and pO2, as well as preventing endotracheal intubation and mortality. The likelihood of requiring switch to an alternative treatment method may be dependent on clinician preference as well as clinical deterioration, although patients are significantly less likely to switch treatment due to interface intolerance.

While there is currently insufficient high‐quality evidence to support the incorporation of HFNC into guidelines for management of AHRF, cases such as those presented by Millar et al.^42^ and Mittal et al.^48^ could suggest the utility of HFNC as a final exhaustive measure in the management of AHRF where NIV or intubation are not indicated. More research is needed to evaluate the effect of HFNC in the management of AHRF associated with conditions other than COPD. More RCTs, longer follow up, larger sample sizes, and appropriate controlling for adjunctive therapies and lack of blinding is required to support the potential use of HFNC for this purpose and possibly justify its inclusion in future clinical guidance for the management of AHRF in practice.

## AUTHOR CONTRIBUTIONS

**Aisling C. Fahey**: Review conception and design, study selection (identification and screening), data collection, quality appraisal and risk of bias assessment, data extraction, data synthesis, data analysis, wrote paper. **Martina O'Connell**: Study selection (identification and screening), quality appraisal and risk of bias assessment, review and approval of paper. **Nicola Cornally**: Supervision, review and approval of paper. **Mohamad M. Saab**: Supervision, review conception and design, data synthesis, data analysis, review and approval of paper.

## CONFLICT OF INTEREST STATEMENT

No conflict of interest has been declared by the authors.

## ETHICS STATEMENT

All analyses were based on previously published results and did not require ethical approval or patient consent.

## Supporting information


**Figure S1.** pCO2 Sensitivity tests.

**Table S1:** Eligibility criteria and associated search terms.

**Table S2:** Data Extraction Table.

**Table S3:** Quality appraisal of randomised controlled trials (n = 7).

**Table S4:** Summary of findings from individual studies.

**Table S5:** Diagnosis & Recommendations.

## Data Availability

The data that supports the findings of this study are available in the supplementary material of this article.
