# Supplementary figures and images for: High flow nasal cannula versus noninvasive ventilation in the treatment of acute hypercapnic respiratory failure: A systematic review and meta‐analysis
Source: Clin Respir J. 2023 Sep 12;17(11):1091–102. doi: 10.1111/crj.13695 (PMC10632084; doi:10.1111/crj.13695)

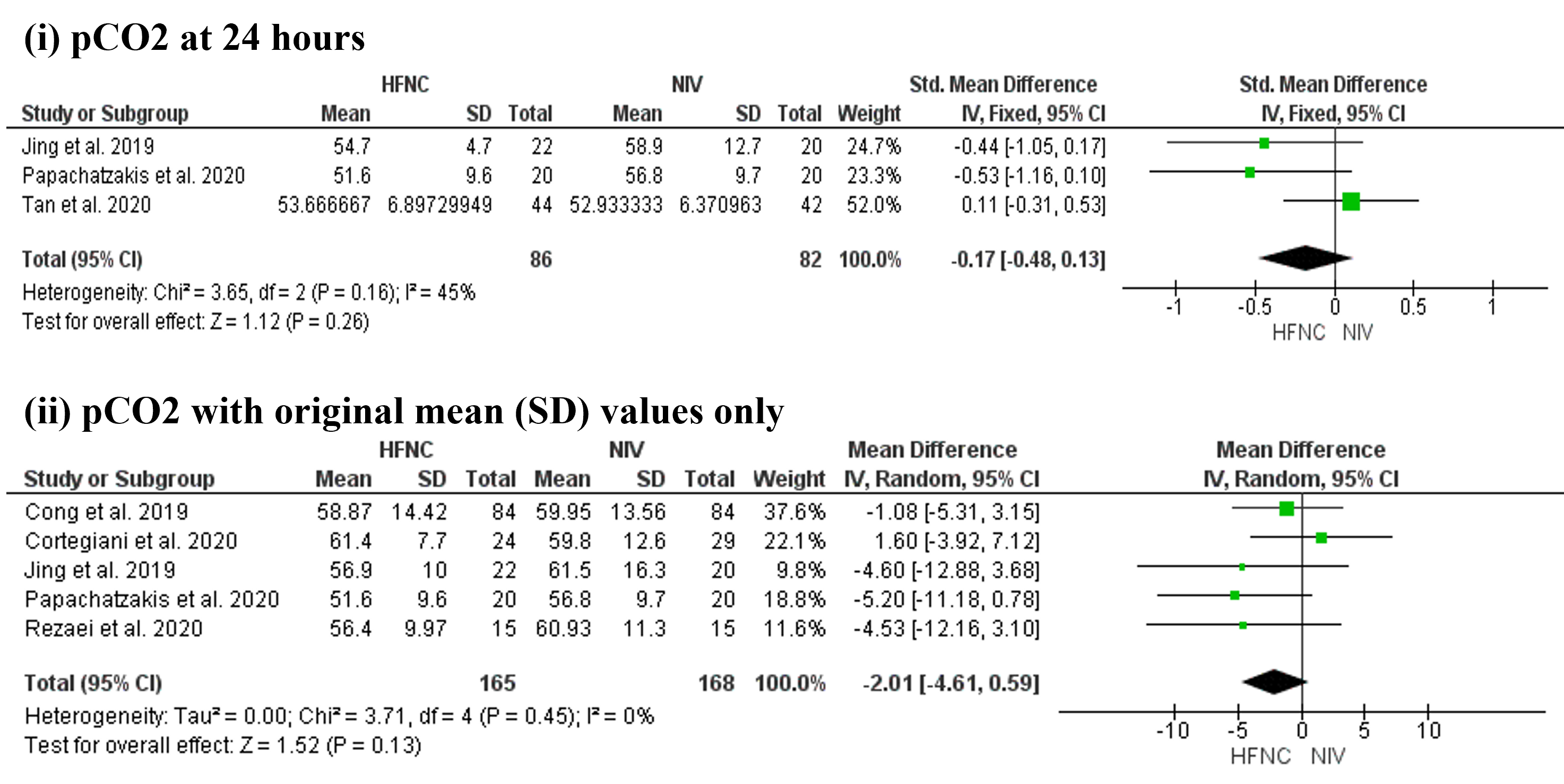

Supplement: Supplementary file 1 — Figure S1. pCO2 Sensitivity tests. [file CRJ-17-1091-s006.png]
