# Supplementary material for: High flow nasal cannula versus noninvasive ventilation in the treatment of acute hypercapnic respiratory failure: A systematic review and meta‐analysis
Source: Clin Respir J. 2023 Sep 12;17(11):1091–102. doi: 10.1111/crj.13695 (PMC10632084; doi:10.1111/crj.13695)
Supplement: Supplementary file 2 — Table S1: Eligibility criteria and associated search terms. [file CRJ-17-1091-s001.docx]

**Supplementary Table 1:** Eligibility criteria and associated search terms

| **PICO framework** | **Inclusion criteria** | **Exclusion criteria** | **Search terms** |
| --- | --- | --- | --- |
| **Population** | Adult patients (≥18 years)  Patients diagnosed with *acute* hypercapnic respiratory failure | Age <18 years  Patients with other conditions requiring invasive/non-invasive ventilation that are not AHRF  Patients with chronic or stable (i.e., non-acute) hypercapnic respiratory failure | hypercapn* OR "type 2 respiratory failure" OR "respiratory acid*" OR AHRF OR (exacerbation N3 “chronic obstructive pulmonary disease*”) OR (exacerbation N3 COPD) OR AECOPD |
| **Intervention** | Experimental or observational designs using HFNC as an intervention | Does not include HFNC as an intervention  Descriptive design used | "high flow nasal" OR HFNC OR HFNO OR "humidified high flow oxygen" OR AIRVO OR "high flow oxygen therap*" OR HFOT OR "high flow oxygen" OR "high velocity nasal insufflation" OR HVNI OR “nasal high flow” |
| **Comparison** | Includes NIV as a comparator | Does not include NIV as a comparator | NIV OR "noninvasive ventilation" OR BIPAP OR "bilevel positive airway pressure" OR "positive airway pressure" OR EPAP OR "expiratory positive airway pressure" OR PEEP OR "positive end expiratory pressure" OR "noninvasive positive pressure ventilat*" OR NIPPV OR NPPV |
| **Outcomes** | Arterial blood gas values  Intubation rates  Mortality rates  Need to switch to the opposite arm of treatment | No identifiable outcomes | Not specified to maximise retrieval |
