# Supplementary material for: High flow nasal cannula versus noninvasive ventilation in the treatment of acute hypercapnic respiratory failure: A systematic review and meta‐analysis
Source: Clin Respir J. 2023 Sep 12;17(11):1091–102. doi: 10.1111/crj.13695 (PMC10632084; doi:10.1111/crj.13695)
Supplement: Supplementary file 3 — Table S2: Data Extraction Table. [file CRJ-17-1091-s005.docx]

**Supplementary Table 2:** Data Extraction Table

| **Author(s), Year, Country** | **Aims & Objectives** | **Design/ Methods** | **Sample/ setting** | **Data Collection tools** | **Interventions** | **Relevant outcome** | **Limitations/ Risk of Bias** |
| --- | --- | --- | --- | --- | --- | --- | --- |
| Cong et al. (2019); China | To compare the therapeutic effects and nursing outcomes of patients with AECOPD treated with HFNC vs patients treated with NIPPV. | Single-centre, prospective, randomized, controlled,  single-blind study. | n = 168  Intervention = 84  Control = 84  Conducted within ICU.  Patients aged 40-76 years were diagnosed with AECOPD, in the ICU, requiring ventilation support, and were haemodynamically stable.  Similar clinical characteristics and demographics among intervention and control groups. | ABG measurements at 12 hours and 5 days, ventilation support time, hospitalisation days, and complications.  Comfort and nursing satisfaction were also measured using hospital-designed questionnaires. | HFNC n = 84; 37°C & flow rate 30-35L/min.  NIPPV n = 84; ventilated by mouth and nose.  Settings began at:  IPAP: 10cmH2O  EPAP: 5cmH2O Pressures gradually  increased patient adapted.  FiO2 was adjusted to ensure oxygen saturation.  Antibiotics were given based on sputum culture results.  Bronchodilator and glucocorticoids given as required. | HFNC and NIPPV both showed significant improvements in all ABG tensions of interest (pO2, pCo2, pH) and SpO2. No significant difference in the degree of improvement between the intervention and control groups.  Complications had lower incidence in HFNC treatment.  Respiratory support time and hospitalisation days were similar between the two groups.  Patient’s comfort and satisfaction was significantly higher in the HFNC group.  Intervention and control therapies had similar clinical efficacy in the treatment oof patients with AECOPD, however comfort, and nursing satisfaction are higher, and incidence of complications are lower with HFNC. | Randomisation process is not disclosed. Unable to find protocol for clarity.  Single-centre trial - limits generalisability. |
| Cortegiani et al. (2020); Italy | Evaluate the short-term effect of HFNT vs NIV in patients with mild-moderate AECOPD hypercapnia. | Multicentre Non-inferiority  Prospective  RCT. | n = 79  Intervention = 40  Control = 39  ED, ICU, Respiratory unit of each hospital.  Participants >18 years; COPD, admitted with mild-moderate hypercapnic respiratory failure.  pH between 7.25-7.35; pCO2 ≥55mmHg. | Vital parameters, Borg scale, and ABGs evaluated at baseline, 2H and 6H after randomisation. | Continuous HFNC or NIV.  HFNT n = 40; setting 60L/min & 37°C to start.  NIV n = 39  EPAP 3-5cmH2O  IPAP to target TV of 6-8mL/kg-1 of ideal body weight. | Both groups significantly lowered pCO2 levels.  HFNT did not cross non-inferiority threshold.  Poor treatment tolerance observed within the NIV group.  HFNT non-inferior to NIV as initial ventilatory support in mild-moderate AECOPD.  HFNT able to significantly reduce pCO2 but 32% of patients switch to NIV by 6H. | Unblinded.  TV was not measured in NIV group; harder to determine if level of pressure support affected comfort levels and tolerance.  EPAP administered was higher than stated in protocol. |
| Doshi et al. (2020); United States of America | To evaluate the response of individuals with hypercapnic RF to HVNI compared to NIV in terms of alleviating hypercapnia and acidosis. | Subgroup analysis of multicentre non-inferiority design RCT comparing HVNI and NIV in all treatment of non-specific respiratory failure. | Subgroup sample n = 65  HVNI n = 34  NIV n = 31  Performed at 2 academic and 3 community centres in US.  Included patients with AECOPD or acute hypercapnic RF.  Emergency Department presentations. | Vital parameters, Borg scale, and ABGs evaluated at baseline, 30, 60, 90 & 240 minutes**.** | Continuous HFNC or NIV.  HVNI: 35-37°C/35 L/min/100% FiO2  n = 34  NIV: IPAP 10-20/EPAP 5-10/FiO2 100%  n = 31 | 50% failure in both groups due to failure to ventilate.  Other 50% failure of NIV was due to tolerance. This did not occur with HVNI.  LOS not statistically different. Slightly shorter ICU LOS for HVNI. | Subgroup analysis of a study answering a different question.  Justification of physician’s decision to crossover not available.  Study sponsored by a HVNI medical device manufacturer who participated in study design/selection & management. |
| Jing et al. (2019); China | To test the hypothesis that HFNC is non-inferior to NIV in maintaining vital signs and ABGs in individuals with COPD who have persistent post-extubation hypercapnia.  Secondary outcomes measured: patient comfort, need for bronchoscopy, use of pulmonary medications, and chest physiotherapy. | Single-centre RCT. Non-inferiority design. | n = 42  HFNC n = 22  NIV n = 20  Patients with COPD intubated due to exacerbation, with hypercapnia (pCO2 >45mmHg) at time of extubation.  Patients also had to meet the Collaborating Research Group for Non-invasive Mechanical Ventilation of the Chinese Thoracic Society (2005) “Pulmonary Infection Control Window” criteria. | Vital signs and ABGs at 3H, 24H & 48H. | HFNC or NIV for at least 8H/day.  HFNC: median flow 50 L/min/37°C/FiO2 titrated to achieve SpO2 88-92%  NIV: Starting pressures  IPAP 10-12cmH2O  EPAP 4-5cmH2O  Subsequent titration of pressures guided by ABGs.  Titrate FiO2 to achieve SpO2 88-92%  Both interventions used for at least 8 hours/day in the 48 hours post extubation. | No significant differences in  mortality in 28 days.  Pressure sore occurrence:  HFNC group n = 0  NIV group n = 4  Comfort scores:  HFNC group were better and fewer patients needed bronchoscopy for secretion management within 48H after extubation.  Post-extubation respiratory failure occurrence:  HFNC: n = 3; (required rescue NIV & did not require reintubation n = 1; Reintubated n = 2).  NIV: n = 1 (reintubated)  HFNC was noninferior to NIV in weaning COPD from invasive ventilation in terms of vital signs and ABGs. | Single centre trial with small sample size.  Baseline pulmonary function of the sample was unknown, so relationship between COPD patients’ pulmonary function status and HFNC success in weaning is not fully known.  Patient comfort measurement did not specifically address dyspnoea, so dyspnoea measurement is unknown. |
| Papachatzakis et al. (2020); Greece | To compare the efficacy of HFNC vs NIV in the management of acute hypercapnic respiratory failure. | Single-centre RCT. | n = 40  Emergency Dept presentations. Moved to HDU for study.  pCO2 >45mmHg  pH <7.20 excluded  Any-causes of AHRF included. | Vital signs and ABGs at baseline, 24H and at discharge.  Comfort and tolerability questionnaire administer to HFNC group. | HFNC or NIV.  HFNC n = 20  35L/min titrated up if tolerated to 45-50L/min.  Maintain SpO2 >90%.  NIV n = 20  BIPAP S/T with gradually increased pressures to maximum tolerated over 1H. | LOS, severe complications, and mortality not significantly different between groups.  n = 3 for NIV crossover to HFNC (due to discomfort, nasal ulcer, lack of cooperation)  HFNC led to significant decrease in pCO2, showed similar in-hospital severe complications and mortality rates compared to NIV.  HFNC was more comfortable and well tolerated. | Single centre study.  Small sample size. |
| Rezaei et al. (2020); Iran | To compare the efficacy of HFNC to NIV in the treatment of COPD during exacerbation | Single centre, randomised clinical crossover trial. | n = 30  Group A (HFNC first) n = 15  Group B (NIV first) n = 15  Emergency Department referrals & ward admissions.  Moderate to severe COPD exacerbation and acute respiratory failure.  pH 7.25-7.35, pCO2 ≥45 mm Hg.  There was no significant difference in the baseline characteristics. | ABGs, respiratory  rate, dyspnoea score, heart rate, SpO2 compared before and after the intervention and between groups. | HFNC or NIV for 30 minutes, followed by 1H washout period, then switching to the other intervention for 30 mins  Group A:  HFNC at 15-35 L/min/37°C, then switched to NIV after washout period.  Group B:  VPAP, then switched to HFNC after washout period. | This study showed that both treatment options, including high-pressure oxygen therapy and non-invasive oxygen delivery, are efficient enough for COPD exacerbation.  Suggests that HFT is non-inferior to NIV in improving patient condition regarding RR, HR, pCO2, and O2 saturation. | Some historical information such as FEV1, home oxygen therapy, pulmonary artery pressure, and the exact cause of exacerbation were not determined.  The patient comfort score was also not evaluated. |
| Tan et al. (2020); China | To test if HFNC is non-inferior to NIV in preventing post-extubation treatment failure in patients with COPD previously intubated for hypercapnic respiratory failure. | Multicentre, unblinded, non-inferiority RCT. | n = 86  HFNC n = 44  NIV n = 42  Within two ICUs.  Patients with COPD and hypercapnic respiratory failure who were already receiving invasive ventilation were randomised to HFNC or NIV at extubation.  ≤ 85 years of age, able to care for themselves within the past year, respiratory failure induced by broncho-pulmonary infection, and meeting the Collaborating Research Group for Non-invasive Mechanical Ventilation of the Chinese Thoracic Society (2005) “Pulmonary Infection Control Window” criteria. | Vitals and ABGs at 1H, 24H & 48H. | Continuous HFNC or NIV for at least two hours. Intermittent usage as required after initial two-hour period.  NIV: S/T mode, EPAP: 4cmH2O initially  IPAP: 8cmH2O  FiO2 adjusted to maintain a respiratory rate ≤ 28/min, SpO2 88–92%, and pCO2 of 45–60mmHg or the last pCO2 level recorded prior to extubation.    HFNC group: 50L/min and adjusted according to patient tolerance, 37 °C, FiO2 was adjusted to maintain an SpO2 of 88–92%. | HFNC after extubation did not have increased rates of treatment failure and had better tolerance and comfort compared to NIV.  Supports the use of HFNC in such patients, especially for those who cannot tolerate NIV.  Intubation rate in the HFNC group was similar to that of the NIV group and treatment switch rate was lower than that in the NIV.  However, there were no significant differences between the two groups in intubation or treatment switch rate.  Intolerance was significantly lower in the HFNC group than in the NIV group. | Small sample size.  Subjective selection of ventilatory support settings. Recommend HFNC gas flow could be titrated through diaphragmatic potential or ultrasound assessment of diaphragmatic muscle movement for better standardisation.  Attending physicians could not be blinded to study group. |
| **Abbreviations:** ABG = arterial blood gas; AECOPD = acute exacerbation of chronic obstructive pulmonary disease; BIPAP = bilevel positive airway pressure; COPD = chronic obstructive pulmonary disease; cmH2O = centimetres of water; ED = emergency department; EPAP = expiratory positive airway pressure; FiO2 = fraction of inspired oxygen; H = hours; HFNC = high flow nasal cannula; HFNT = high flow nasal therapy; HR = heart rate; HVNI = high velocity nasal insufflation; ICU = intensive care unit; IPAP = inspiratory positive airway pressure; KG = kilograms; LOS = length of stay; L/min = litres per minute; mmHg = millimetres of mercury; n = sample size; NIV = non-invasive ventilation; NIPPV = non-invasive positive pressure ventilation; O = objectives; pCO2 = partial pressure of carbon dioxide; pO2 = partial pressure of oxygen; RCT = randomised controlled trial; RF = respiratory failure; RR = respiratory rate; SpO2 = saturation of peripheral oxygen; S/T = spontaneous/timed; TV = tidal volume; VPAP = variable positive airway pressure. | | | | | | | |
