# Supplementary material for: High flow nasal cannula versus noninvasive ventilation in the treatment of acute hypercapnic respiratory failure: A systematic review and meta‐analysis
Source: Clin Respir J. 2023 Sep 12;17(11):1091–102. doi: 10.1111/crj.13695 (PMC10632084; doi:10.1111/crj.13695)
Supplement: Supplementary file 4 — Table S3: Quality appraisal of randomised controlled trials (n = 7). [file CRJ-17-1091-s003.docx]

**Supplementary Table 3:** Quality appraisal of randomised controlled trials (n = 7)

| JBI Items (Randomised Controlled Trial) | Cong et al. (2019) | Cortegiani et al. (2020) | Doshi et al. (2020) | Papachatzakis et al. (2020) | Jing et al. (2019) | Rezaei et al. (2020) | Tan et al. (2020) |
| --- | --- | --- | --- | --- | --- | --- | --- |
| 1. Was true randomization used for assignment of participants to treatment groups? | Unclear | Yes | Yes | Unclear | Yes | Yes | Yes |
| 1. Was allocation to treatment groups concealed? | Unclear | Yes | Yes | Unclear | Unclear | Yes | Yes |
| 1. Were treatment groups similar at the baseline? | Yes | Yes | No† | Yes | Yes | Yes | Yes |
| 1. Were participants blind to treatment assignment? | N/A | N/A | N/A | N/A | N/A | N/A | N/A |
| 1. Were those delivering treatment blind to treatment assignment? | N/A | N/A | N/A | N/A | N/A | N/A | N/A |
| 1. Were outcomes assessors blind to treatment assignment? | Unclear | Yes | Unclear | Unclear | Unclear | Yes | Yes |
| 1. Were treatment groups treated identically other than the intervention of interest? | Yes | Unclear | Unclear | Unclear | Yes | Yes | Unclear |
| 1. Was follow up complete and if not, were differences between groups in terms of their follow up adequately described and analyzed? | Yes | Yes | N/A | Unclear | Yes | Yes | Yes |
| 1. Were participants analyzed in the groups to which they were randomized? | Yes | Yes | Yes | Yes | Yes | Yes | Yes |
| 1. Were outcomes measured in the same way for treatment groups? | Yes | Yes | Yes | Yes | Yes | Yes | Yes |
| 1. Were outcomes measured in a reliable way? | Yes | Yes | Yes | Yes | Yes | Yes | Yes |
| 1. Was appropriate statistical analysis used? | Yes | Yes | Yes | Yes | Yes | Yes | Yes |
| 1. Was the trial design appropriate, and any deviations from the standard RCT design (individual randomization, parallel groups) accounted for in the conduct and analysis of the trial? | Yes | Yes | Yes | Yes | Yes | Yes | Yes |

† Not statistically significant
