# Supplementary material for: High flow nasal cannula versus noninvasive ventilation in the treatment of acute hypercapnic respiratory failure: A systematic review and meta‐analysis
Source: Clin Respir J. 2023 Sep 12;17(11):1091–102. doi: 10.1111/crj.13695 (PMC10632084; doi:10.1111/crj.13695)
Supplement: Supplementary file 5 — Table S4: Summary of findings from individual studies. [file CRJ-17-1091-s002.docx]

**Supplementary Table 4:** Summary of findings from individual studies

| **Author(s), year & country** | **Findings**† |
| --- | --- |
| Cong et al. (2019); China | **O1.**   \|  \| Mean (SD) \| \| \| \| \| \| \| --- \| --- \| --- \| --- \| --- \| --- \| --- \| \| Timepoint \| pH \| \| pCO2 (mmHg) \| \| pO2 (mmHg) \| \| \|  \| HFNC \| NIV \| HFNC \| NIV \| HFNC \| NIV \| \| End point of study (5 days) \| 7.35 (0.08) \| 7.36 (0.07) \| 58.87 (14.42) \| 59.95 (13.56) \| 81.87 (15.27) \| 82.22 (15.64) \| \| 24H (if reported) \| NR \| NR \| NR \| NR \| NR \| NR \|   **O2.** Not reported.  **O3.** Not reported.  **O4.** Not reported. |
| Cortegiani et al. (2020); Italy | **O1.**  HFNC was statistically non-inferior to NIV as initial ventilatory support in decreasing pCO2 after 2H of treatment in patients with mild-to-moderate AECOPD. However, 32% of patients receiving HFNC required NIV by 6H.   \|  \| Mean (SD) \| \| \| \| \| \| \| --- \| --- \| --- \| --- \| --- \| --- \| --- \| \| Timepoint \| pH \| \| pCO2 (mmHg) \| \| pO2 (mmHg) \| \| \|  \| HFNC \| NIV \| HFNC \| NIV \| HFNC \| NIV \| \| End point of study (6H) \| NR \| NR \| 61.4 (7.7) \| 59.8 (12.6) \| NR \| NR \| \| 24H (if reported) \| NR \| NR \| NR \| NR \| NR \| NR \|   **O2.** n = 2/40 patients (5%) of HFNC group and n = 1/39 (2.6%) of NIV group underwent intubation during hospitalisation.  **O3.** In hospital mortality n = 2/40 (5%) in HFNC group and n = 6/39 (16.4%) in NIV group.  **O4.** Treatment switch from baseline to 6H: HFNC: n = 13/40 patients (32.5%), of which 5 were intolerant, remaining 8 secondary to worsening condition/no improvement; NIV: n = 3/39 patients (7.7%), of which 3 intolerant; Treatment switch *p* = 0.0061.  A higher proportion of patients in the NIV group showed poor tolerance to the intervention by 6H n = 29/39 (74%) compared to HFNC n = 14/40 (35%); *p* = 0.0019. |
| Doshi et al. (2020); United States | **O1.** The primary finding of this study is that HFNC can provide ventilatory support that is of comparable efficacy to NIV in the setting of acute hypercapnic respiratory failure.   \|  \| Median [IQR] \| \| \| \| \| \| \| --- \| --- \| --- \| --- \| --- \| --- \| --- \| \| Timepoint \| pH \| \| pCO2 (mmHg) \| \| pO2 (mmHg) \| \| \|  \| HFNC \| NIV \| HFNC \| NIV \| HFNC \| NIV \| \| End point of study (240 mins) \| 7.38 [7.34-7.42] \| 7.35 [7.33-7.37] \| 50 [42-58] \| 57 [47-70] \| 83 [40-99] \| 88 [77-97] \| \| 24H (if reported) \| NR \| NR \| NR \| NR \| NR \| NR \|   **O2.** 72H intubation: HFNC: n = 2/34 (5.9%) and NIV: n = 5/31 (16.1); *p* = 0.2443.  **O3.** Not reported.  **O4.** 72H treatment switch: HFNC: n = 8/34 (23.5%) and NIV: n = 4/31 (12.9%); *p* =0.346.  In the HFNC group, no patient failed treatment due to an inability to tolerate the therapy.  Of the NIV group that failed secondary to intolerance, 3 of the 4 were managed successfully with HFNC, suggesting that at least in patients that do not seem to tolerate NIV, HFNC may be a reasonable option prior to intubation. |
| Jing et al. (2019); China | **O1.**   \|  \| Mean (SD) \| \| \| \| \| \| \| --- \| --- \| --- \| --- \| --- \| --- \| --- \| \| Timepoint \| pH \| \| pCO2 (mmHg) \| \| pO2 (mmHg) \| \| \|  \| HFNC \| NIV \| HFNC \| NIV \| HFNC \| NIV \| \| End point of study (48H) \| 7.43 (0.07) \| 7.41 (0.06) \| 56.9 (10.0) \| 61.5 (16.3) \| NR \| NR \| \| 24H (if reported) \| 7.46 (0.03) \| 7.42 (0.05) \| 54.7 (4.7) \| 58.9 (12.7) \| NR \| NR \|   **O2.** HFNC reintubated n = 2/22 (9.1%); NIV reintubated n = 1/20 (5.0%)  **O3.** All‐cause mortality in 28 days: HFNC group n = 5 /22 (22.7%); NIV group n = 5/20 (25%); not statistically significant.  **O4.** Not reported. |
| Papachatzakis et al. (2020); Greece | **O1.**   \|  \| Mean (SD) \| \| \| \| \| \| \| --- \| --- \| --- \| --- \| --- \| --- \| --- \| \| Timepoint \| pH \| \| pCO2 (mmHg) \| \| pO2 (mmHg) \| \| \|  \| HFNC \| NIV \| HFNC \| NIV \| HFNC \| NIV \| \| End point of study (24H) \| 7.4 (0.1) \| 7.4 (0.1) \| 51.6 (9.6) \| 56.8 (9.7) \| 67.9 (8.8) \| 72.0 (10.4) \|   **O2.** No patients required intubation.  **O3.** Mortality rates were equal in both groups. n = 20 in both groups; mortality n = 3/20 in both groups (accounting for 15% of patients in each).  **O4.** HFNC was comfortable and well tolerated, as none of the patients in this group changed from HFNC to NIV; 3 patients (7.5% of total sample) from the control group changed from NIV to HFNC due to discomfort, nasal ulcer, and lack of cooperation; *p* = 0.608. |
| Rezaei et al. (2020); Iran | **O1.**   \|  \| Mean (SD) \| \| \| \| \| \| \| --- \| --- \| --- \| --- \| --- \| --- \| --- \| \| Timepoint \| pH \| \| pCO2 (mmHg) \| \| pO2 (mmHg) \| \| \|  \| HFNC \| NIV \| HFNC \| NIV \| HFNC \| NIV \| \| End point of study (6H) \| 7.32 (0.03) \| 7.34 (0.02) \| 56.4 (9.97) \| 60.93 (11.3) \| NR \| NR \| \| 24H (if reported) \| NR \| NR \| NR \| NR \| NR \| NR \|   After the second intervention, ABG indexes were on the same level, but the patients’ Borg scale in group B (started with NIV) was significantly better than that in group A (started with HFNC).  **O2.** Not reported.  **O3.** Not reported.  **O4.** Not reported. |
| Tan et al. (2020); China | **O1.**   \|  \| Median [IQR] \| \| \| \| \| \| \| --- \| --- \| --- \| --- \| --- \| --- \| --- \| \| Timepoint \| pH \| \| pCO2 (mmHg) \| \| pO2 (mmHg) \| \| \|  \| HFNC \| NIV \| HFNC \| NIV \| HFNC \| NIV \| \| End point of study (48H) \| 7.43 [7.41–7.49] \| 7.45 [7.41–7.48] \| 51 [49–57.8] \| 52 [49–56] \| NR \| NR \| \| 24H (if reported) \| 7.44 [7.41–7.48] \| 7.45 [7.41–7.48] \| 54 [49–58] \| 52.5 [49–57.3] \| NR \| NR \|   The *p*-value comparison of pH and pCO2 change from HFNC were statistically significant (0.002 & 0.016, respectively).  NIV findings within these parameters were not statistically significant.  **O2.** Invasive ventilation: HFNC: n = 6/44 (13.6%); NIV: n = 6/42 (14.29%); *p* = 0.931.  **O3.** 28-day mortality: HFNC: n = 7/44 (15.9%); NIV: n = 5/42 (11.9%); *p* = 0.758.  **O4.** Treatment switch: HFNC: n = 4/44 (9.1%); NIV: n = 6/42 (14.3%); *p* = 0.516.  Treatment intolerance: HFNC: n = 0/10 failures (0%); NIV: n = 6/12 failures (50.0%); *p* = 0.015.  The causes for six intolerances in the NIV group were feelings of claustrophobia (n = 2), excessive air flow or pressure (n = 2), breathlessness (n = 1), and headache (n = 1).  Reasons for failure:  Aggravation of respiratory distress: HFNC: n = 5/10 (50%); NIV: n = 2/12 (16.67%); *p* = 0.172.  Aggravation of hypoxemia: HFNC: n = 2/10 (20%); NIV: n = 1/12 (8.33%); *p* = 0.571.  Aggravation of carbon dioxide retention: HFNC: n = 3/10 (30%); NIV: n = 3/12 (25%); *p* = 1.0. |
| **†Outcomes (O):** O1 = ABG values; O2 = Intubation; O3 = Mortality; O4 = Treatment switch  **Abbreviations:** ABG = arterial blood gas; AECOPD = acute exacerbation of COPD; H = hours; HFNC = high flow nasal cannula; IQR = interquartile range; mmHg = millimetres of mercury; n = sample size; NIV = non-invasive ventilation; NR = not reported; O = outcome; pCO2 = partial pressure of carbon dioxide; pO2 = partial pressure of oxygen; SD = standard deviation | |
