# Supplementary material for: High flow nasal cannula versus noninvasive ventilation in the treatment of acute hypercapnic respiratory failure: A systematic review and meta‐analysis
Source: Clin Respir J. 2023 Sep 12;17(11):1091–102. doi: 10.1111/crj.13695 (PMC10632084; doi:10.1111/crj.13695)
Supplement: Supplementary file 6 — Table S5: Diagnosis & Recommendations. [file CRJ-17-1091-s004.docx]

**Supplementary Table 5:** Diagnosis & Recommendations

| **Author** | **Postextubation** | **COPD** | **CPE/CHF** | **OSA/OHS** | **PE** | **Pneumonia** | **Miscellaneous: Neuromuscular, Bronchiectasis, Asthma, Unspecified** | **Acidosis** | **Recommendations** | **Further study required** |
| --- | --- | --- | --- | --- | --- | --- | --- | --- | --- | --- |
| Cong, et al. (2019) | N | Y | N | N | N | N | N | N | Both effective methods for treating AHRF. | N |
| Cortegiani, et al. (2020) | N | Y | N | N | N | N | N | Y | Non-inferior to NIV in mild-moderate AHRF | Y |
| Doshi, et al. (2020) | N | Y | N | N | N | N | Y | Y | Potential use in AHRF | Y |
| Jing, et al. (2019) | Y | Y | N | N | N | N | N | N | May be useful in weaning individuals with COPD post-extubation for AHRF | Y |
| Papachatzakis, et al. (2020) | N | Y | Y | N | N | N | N | N | Lower PCO2 in HFNC groups suggests HFNC was superior.  May be an alternative to NIV if NIV is poorly tolerated | Y |
| Rezaei, et al. (2020) | N | Y | N | N | N | N | N | Y | Could be a possible treatment option in the emergency department for moderate to severe exacerbations of COPD with AHRF | Y |
| Tan et al. (2020) | Y | Y | N | N | N | N | N | N | In patients with COPD and severe hypercapnic respiratory failure who received invasive ventilation, HFNC may be used, especially when NIV is poorly tolerated. | Y |

**Key:** Y = present in this paper; N = not present in this paper

**Abbreviations:** AHRF = acute hypercapnic respiratory failure; CPE = cardiogenic pulmonary oedema; COPD = chronic obstructive pulmonary disease; HFNC = high flow nasal cannula; NIV = non-invasive ventilation; PCO2 = partial pressure of carbon dioxide.
